# Acoustic Emission Analysis of Corroded Reinforced Concrete Columns under Compressive Loading

**DOI:** 10.3390/s20082412

**Published:** 2020-04-23

**Authors:** Qiang Li, Xianyu Jin, Dan Wu, Hailong Ye

**Affiliations:** 1 College of Civil Engineering and Architecture, Zhejiang University of Water Resources and Electric Power, Hangzhou 310018, China; liq@zjweu.edu.cn; 2 Department of Civil Engineering, Zhejiang University, Hangzhou 310027, China; xianyu@zju.edu.cn (X.J.); wudan913@zju.edu.cn (D.W.); 3 Department of Civil Engineering, The University of Hong Kong, Pokfulam, Hong Kong, China

**Keywords:** reinforced concrete column, corrosion, acoustic emission, failure analysis, structural mechanics

## Abstract

In this work, the failure process of non-corroded and corroded reinforced concrete (RC) columns under eccentric compressive loading is studied using the acoustic emission (AE) technique. The results show that reinforcement corrosion considerably affects the mechanical failure process of RC columns. The corrosion of reinforcement in RC columns leads to highly active AE signals at the initial stage of loading, in comparison to the non-corroded counterparts. Also, a continuous AE hit pattern with a higher number of cumulative hits is observed for corroded RC columns. The spatial distribution and evolution of AE events indicate that the reinforcement corrosion noticeably accelerates the initiation and propagation of cracking in the RC columns during compressive loading. The AE characteristics of corroded RC columns are in agreement with the macroscopic failure behaviors observed during the damage and failure process. A damage evolution model of corroded RC columns based on the AE parameters is proposed.

## 1. Introduction

Corrosion of steel is the major cause of deterioration in the serviceability and sustainability of reinforced concrete (RC) structures, especially for those in the chloride-laden environment [1,2,3]. As steel corrosion propagates, the volumetric expansion due to rust formation generates expansive pressure on the surrounding concrete and eventually leads to cracking and peeling-off of concrete cover [4,5,6,7,8]. In addition, the bond between steel bars and surrounding concrete degrades due to the formation of corrosion-induced interfacial cracks [9]. Previous investigations regarding the corrosion-induced concrete cover cracking, as well as the influence of cracks on the acceleration of structural deterioration, has been extensively conducted [3,10,11,12]. However, to accurately assess and predict the service life performance of RC structures, understanding the influence of rebar corrosion on the mechanical failure of structural members is crucial. Serving as the vertical bearing member, the column is an important component in the RC structures, as emphasized by the general structural design concept of “strong column, weak beam” principle. Therefore, studies on the internal damage evolution, failure process, and mechanical properties of corroded RC columns are intriguing.

Many efforts have been made on evaluating the mechanical performance of corroded RC columns [13,14,15,16,17]. For instance, Tapan et al. [13] investigated the effects of reinforcement corrosion and peeling-off of concrete cover on the mechanical degradation of RC columns. Wang et al. [14] studied the mechanical behaviors of RC columns with localized corrosion using the accelerated corrosion methods. Most previous experimental tests were destructive in terms of obtaining the ultimate load-bearing capacity. The process of damaging and cracking RC columns is not clearly revealed by the destructive testing methods. Although the destructive methods can obtain the ultimate bearing capacity, deflection, stress-strain curve, crack width, failure mode, and other macroscopic mechanical properties, they fail to obtain the internal damage parameters of RC members under external loading [18].

Acoustic emission (AE) is a transient elastic wave generated due to the rapid release of energy from sources within a material or structure [19,20], which allows continuous detection and sensing of cracking and damage. Although the damage process of RC structures is highly complex, the AE phenomenon during the development of damage and fracture still exists [21]. In 1960, Rüsch [22] studied the AE signal of concrete under loading. In 1970, Wells [23] developed an instrument that records the AE signals of deformed concrete. Thanks to the great advance in AE technology over decades, many scholars have used it to study the damage process, deteriorating mechanisms, and failure process of many physical-chemical-thermal-mechanical processes in concrete structures and materials [21,24,25,26,27]. For instance, Ohtsu summarized the AE characteristics of concrete materials [28,29]. In addition, experimental investigations on AE characteristics and failure mechanisms of RC beams were carried out [30,31,32]. The load-bearing capacity of RC beams was characterized and assessed based on the AE as well [33,34]. These studies have made great contributions to our understanding of the mechanism and failure process of RC members. 

However, most previous studies using AE primarily are limited to the non-corroded flexural RC members. There are several fundamental differences in terms of mechanical responses between a flexural member (e.g. beam) and a compressive member (e.g. column). Also, the in-service RC structures under loading are constantly subjected to steel corrosion [35,36]; as such, the mechanical properties and AE parameters of non-corroded RC members do not accurately represent the actual working status of most RC members [37]. There is no doubt that the corrosion of reinforcement has considerable impacts on the degradation mechanism, deterioration process, and durability of RC structures. Although the previous research on compressive members [38,39] and the corrosion process in RC members [40,41,42] using the AE technique do exist, the use of AE technique in investigating the damage process of corroded RC columns is rarely documented. 

This paper aims to study the AE characteristics and space–time evolution of the corroded RC columns subjected to eccentric compression. Also, a damage evolution model of RC columns based on the AE parameters is proposed based on the test results. 

## 2. Experimental Procedures

### 2.1. Material and Specimen Preparation 

The RC columns with an identical dimension of 120 × 120 × 750 mm were studied. The ordinary Portland cement (OPC) (equivalent to ASTM C150 Type I) blended with ground granulated blast-furnace slag (GGBS), river sand with fineness modulus of 2.64, and crushed limestone coarse aggregate with a continuous 5–20 mm gradation were used. The adopted mixture proportion of concrete (by weight %) was binder: water: sand: coarse aggregate = 1.0: 0.53: 2.0: 3.0, as shown in Table 1. In addition, the compressive strength of concrete after 28 days standard moist curing was 29.47 MPa on 100 mm cubic specimens. On the other hand, the longitudinal reinforcements were 10 mm in diameter, using hot-rolled plain steel bar with the characteristic yield strength of 235 MPa, while stirrups were 6 mm in diameter. 

**Figure 1:**
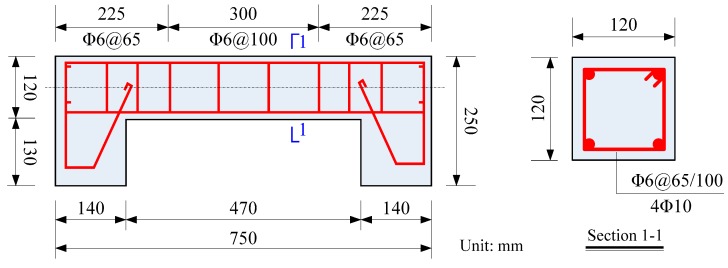
Configuration of the specimen (unit: mm), the thickness of concrete covers was 15 mm.

The specimen configuration with the detailed reinforcement layout is shown in Figure 1. Columns were cast in customized wooden molds. After casting for 24 h, the specimens were demolded and then cured in an environmental chamber with 90% ± 5% relative humidity and 20 ± 2 ℃ for 28 days. 

### 2.2. Corrosion of Specimens

To investigate the influence of corroded reinforcement on the mechanical performance of RC columns, two types of small eccentric columns (e/h = 25/120 mm, where e is the eccentricity, and h is the height of the section) with different corrosion levels were prepared. One was non-corroded while another was partially corroded, both of which had the same configurations and materials. 

**Figure 2:**
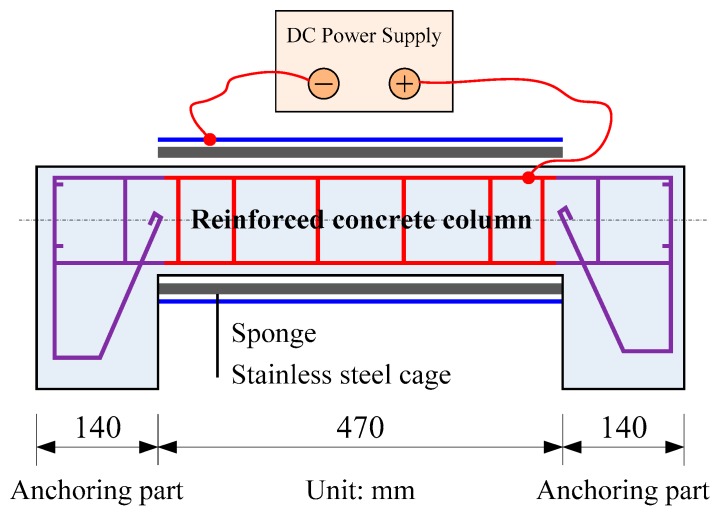
Corrosion setup.

To induce corrosion of reinforcements, the impressed current method was adopted in this study. The setup for accelerating the corrosion process is shown in Figure 2. The surface of the specimen was wrapped by a layer of sponge, followed by the stainless steel cage. To prevent the destruction and corrosion of the anchoring parts, the reinforcements were covered with epoxy resin in the region of 120 mm at both ends. Therefore, the effective corrosion length of reinforcement was 470 mm. The reinforcement and the stainless steel cage wire were connected to a power supply to induce a constant electrical current. The direction of the current was such that the reinforcement served as an anode while the stainless steel wire served as a cathode. To achieve a targeted corrosion rate of approximately 10%, the amount of corrosion was estimated by using Equation (1), according to Faraday’s law [43]. The duration of the corrosion process was 754.48 h with a constant current of 0.2477 A, as shown in Table 2.

(1) $$t=\frac{z_{Fe}FM_{loss}}{M_{Fe}I_{corr}}=\frac{2\times 96500\times M_{loss}}{56\times I_{corr}}\times \frac{1}{3600}=\frac{3446.429}{3600}\frac{M_{loss}}{I_{corr}},$$

where *t* is time (seconds); *M_loss_* is steel mass loss (g); $z_{Fe}$ is ion charge (2 moles of electrons); *F* is Faraday’s constant, which represents the amount of electrical charge in one mole of electron amperes; Icorr is current (amperes); *M_Fe_* is the atomic weight of metal (56 g for Fe).

(2) $$I_{corr}=i_{corr}A_{s,0},$$

where *i_corr_* is the current density, and *A_s,0_* is the surface area of the steel to be rusted. 

### 2.3. Monitoring System during Loading

**Figure 3:**
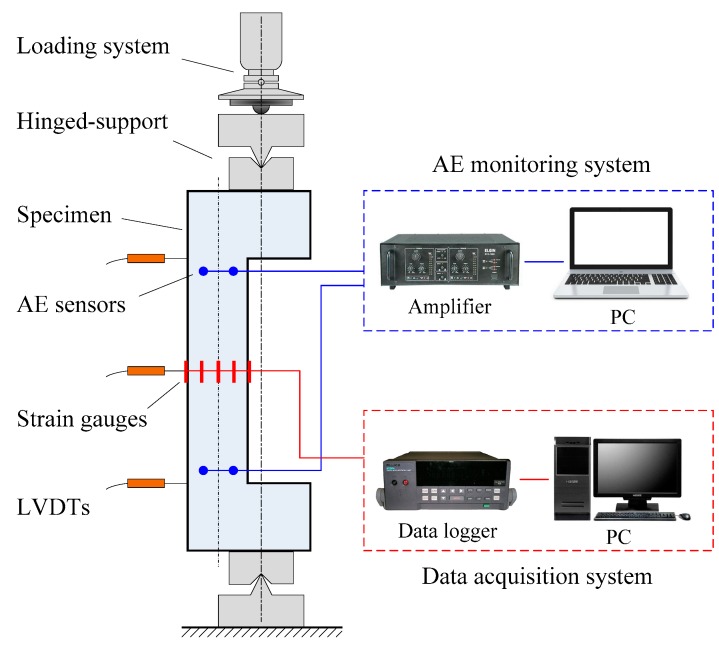
Schematic of the test system. (Abbreviation: acoustic emission (AE))

**Figure 4:**
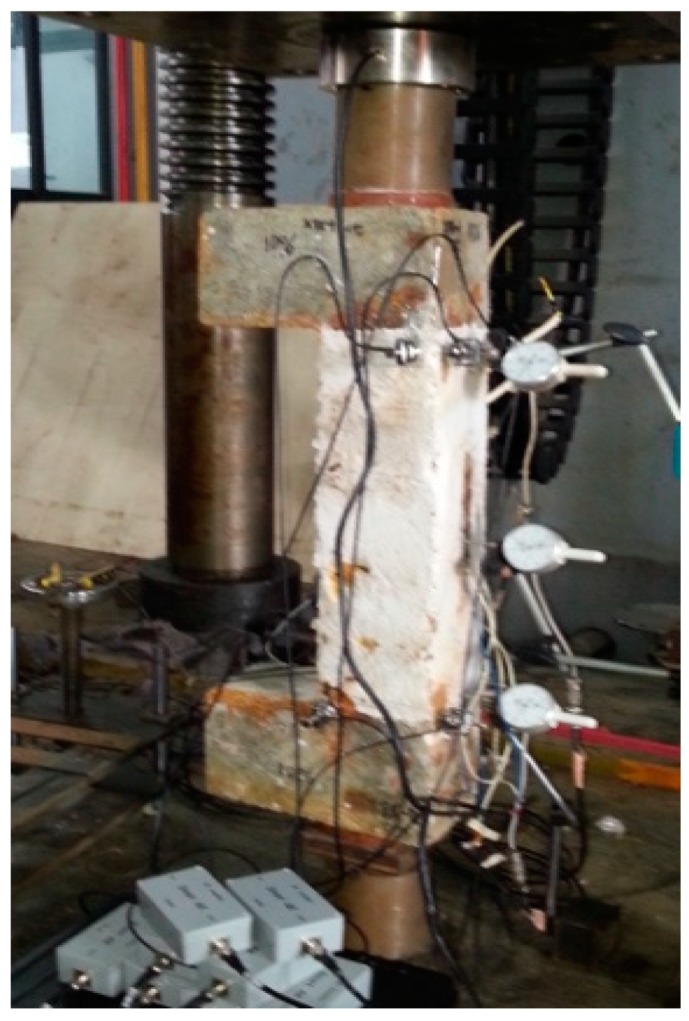
Photograph of the test setup.

**Figure 5:**
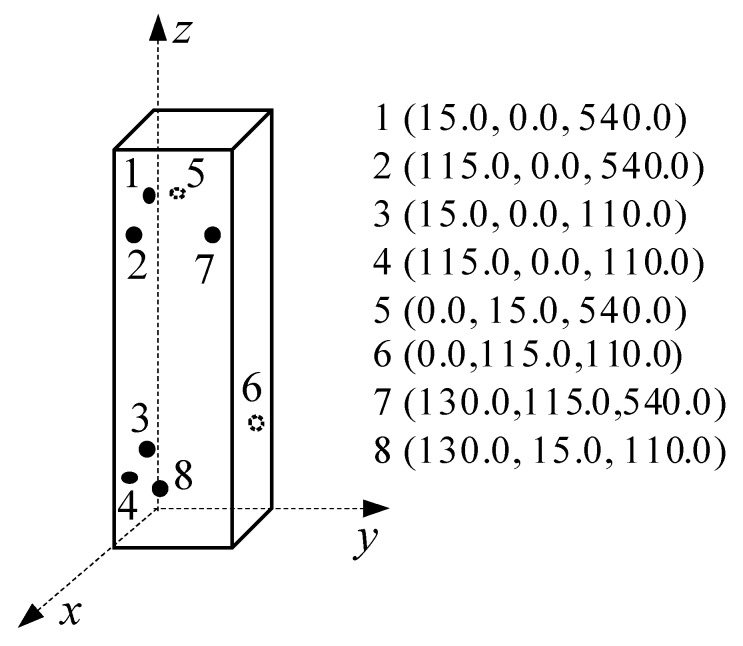
Distribution of AE sensors on specimens.

The monitoring systems including the loading system, AE signal data acquisition system, and the load and displacement recording system are shown in Figure 3 and Figure 4. In particular, the AE acquisition system DS2-8B manufactured by Beijing Science and Technology Company was adopted. Eight RS-35C sensors with a frequency of 150 kHz and 40 dB pre-amplification (parameters suggested in [42]) were used in the AE system. The sampling frequency of the AE acquisition system was 2.5 MHz. To obtain a three-dimensional location of AE events, sensors were attached at the three sides of the columns collecting signals during the whole loading process. Figure 5 shows the spatial arrangement of eight AE sensors on the column specimens. 

**Figure 6:**
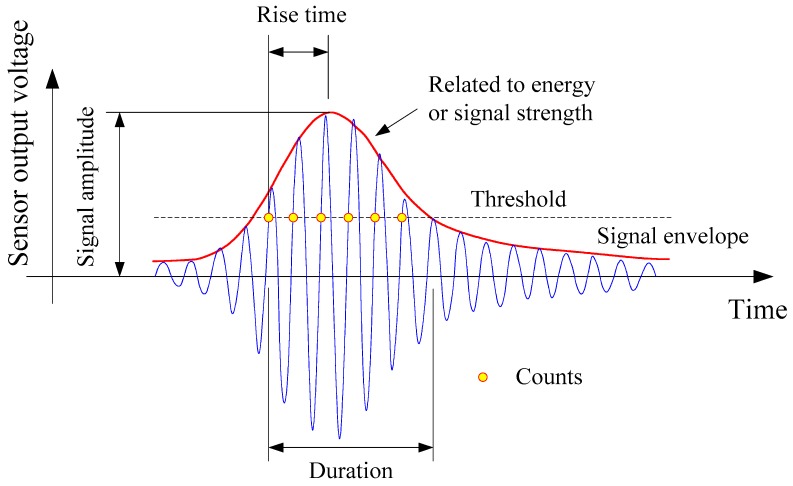
Typical AE signal and its characteristics [44].

The major characteristic parameters of AE include the signal amplitude and energy, hit number, rising time, and duration, with their corresponding physical meanings shown in Figure 6. The parameters can be divided into two categories, namely, intensity and activity. Intensity is used to indicate the strength of the AE signal, mainly characterized by parameters such as amplitude and energy. In general, the greater the amplitude and the higher the energy, the greater the degree of structural damage. While the intensity is used to indicate the frequency of AE signals, reflecting the real-time changes and development of defects, mainly described by parameters such as hit number, hit rate, and cumulative hitting count. The faster the cumulative hitting count growth and the greater the hit rate, the more active the AE signal and the greater the damage development rate. 

The eccentric static loading was applied to the specimens by using the four-post universal testing machine. In the test, the upper end of the column was connected with a ball bearing hinge support, while the lower end of the column was connected with a one-way knife hinge support. Three transducers were arranged on the bending side of the column to measure the lateral deflection. In total five strain gauges were arranged with two on tensile and compressive sides each and another three on the lateral side, as shown in Figure 3. Considering the test characteristic of corroded RC members, the loading interval was less than 20% failure load before specimen cracking and less than 10% cracking load when close to the cracking load, according to the Chinese Standard GBT50152-2012. The load duration per level was not less than 15 minutes. The Donghua DH5937 dynamic strain acquisition and analysis system was used to collect the evolution of load, displacement, and strain. The sampling frequency of the dynamic strain acquisition system was 10 Hz. The specimens were pre-loaded by 2 kN to ensure the reliability of the equipment before data collection. The loading was terminated when the column reached a failure stage.

## 3. Results and Discussion

### 3.1. Corrosion-Induced Crack Behavior

**Figure 7:**
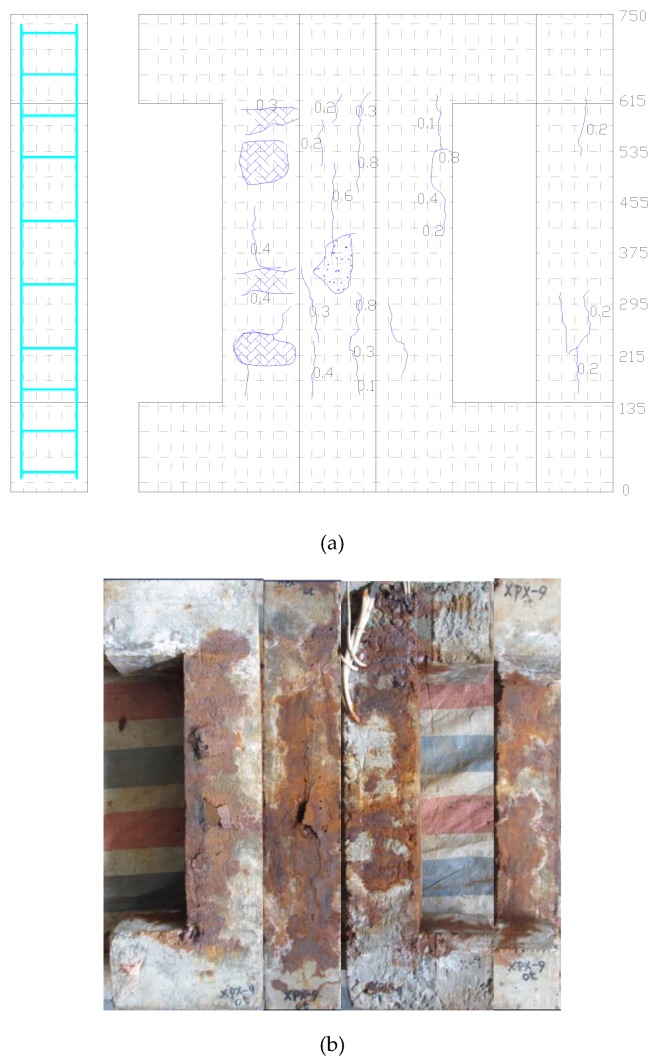
Corrosion-induced crack patterns: (**a**) cracking maps of corroded reinforced concrete (RC) column; (**b**) distribution of cracks and leakage of rust from reinforcement corrosion on the surfaces of the RC column specimens. Unit: mm.

The cracking behavior of the corroded RC column induced by reinforcement corrosion is shown in Figure 7. It can be seen that the stirrup corrosion leads to the bulging of the concrete cover at the location of the stirrup, while the corrosion of the main reinforcement mainly causes the longitudinal cracks. This is mainly because the concrete cover of the stirrup is comparatively thinner, and the stirrups pose certain constraint effects on the longitudinal rust expansion-induced cracking. It is also evidence that the distribution of corrosion products leakage differs in four sides of the column, which could be attributed to the corrosion process, variation of chloride ions and position of the column, and/or other random factors [16].

### 3.2. Corrosion of Reinforcements

To obtain the accurate corrosion rate of the reinforcements embedded in the corroded RC column, the specimen was further broken down and the reinforcement cage in it was taken out after the bearing capacity test. According to the standard ASTM G1-03, the corrosion products were thereafter removed by mechanical and chemical cleaning, the mass of each corroded reinforcement was weighed, and the actual corrosion rate generally defined as the weight loss percentage of the original state was calculated per Equation (3) [45], where ∆*m* is the weight loss, and *m_i_* and *m_f_* are the mass of the reinforcement before and after corrosion, respectively:

(3) $$\eta_{a}=\frac{\Delta m}{m_{i}}\times 100\%=\frac{m_{i}-m_{f}}{m_{i}}\times 100\%.$$

For better illustration, the results expressed as average values of weight loss both for longitudinal steel bars and stirrups are listed respectively in Table 3. Overall, the results indicate that although the average weight losses of reinforcements are approximately close to the targeted rate, the weight loss of stirrups is much higher than that of the longitudinal steel bars, with the actual corrosion rate of the stirrups above the targeted corrosion rate while that of longitudinal steel bars distinctively below, which may mainly be attributed to the following three reasons. First, the concrete cover of the stirrups is relatively thinner compared with the longitudinal steel bars. The corrosion solution therefore first penetrated to the surface of the stirrups and then to that of the longitudinal steel bars. The humidity of the concrete near the surface of the concrete cover was higher, resulting in lowered electrical resistivity that allows for larger currents flowing through the stirrups. The second reason is that the diameter of the longitudinal reinforcements is larger than that of the stirrups, and the current density of the stirrups is greater when the same current flows through the longitudinal reinforcements and the stirrups. According to Faraday’s law, the corrosion of the steel bar is proportional to the current density. Third, the corrosion potential of the stirrups is higher, which plays a role of galvanic protection to the longitudinal steel bars.

### 3.3. Structural Performance of Specimens

#### 3.3.1. General Behaviors

The failure characteristics of the non-corroded column are as follows. The failure first occurred on the compressive side near the axial force; the concrete was crushed and a transverse crack appeared in the concrete on the tensile side at the midspan of the column. When approaching the ultimate bearing capacity, many longitudinal cracks developed rapidly on the lateral side near the axial force. When the ultimate bearing capacity was reached, the crushed concrete burst out suddenly with a loud sound, accompanied by the steel bars that buckled outwards in the shape of a lantern in the corresponding section area. The concrete cover of the longitudinal steel bars was seriously flaked, while a relatively small wedge-shaped spalling of concrete formed on the cover between the longitudinal reinforcements. The failure was sudden and brittle without obvious signs indicated.

It is observed that the primary difference of failure between the corroded column and the non-corroded column is that the concrete cover was merely peeled off in the case of the corroded column. The failure mode of the corroded column is the same as that of the non-corroded one, both of which are characterized by concrete crushing in the pressure zone, and an obvious appearance of a through crack that appeared in the middle part of the tension zone. When the corrosion column was approaching the ultimate load, the majority of originally rust-swelled cracks in the compression zone were further developed and followed by the concrete cover falling off along the cracks, which is different from the failure characteristics of the non-rust eccentric column. Compared with the non-corroded column, there was less concrete crushing and sudden burst, and the accompanying sound was not as significant and crisp as that of the non-corroded eccentric column when it reached the ultimate bearing capacity. Most of the concrete cover was peeled off as a whole spanning 1 to 3 stirrup spacings. The main reason is that the corrosion of the longitudinal steel bars and stirrups results in the rust expansion force and the stirrups can not restrain the concrete cover effectively.

#### 3.3.2. Load-Vertical Displacement Response

**Figure 8:**
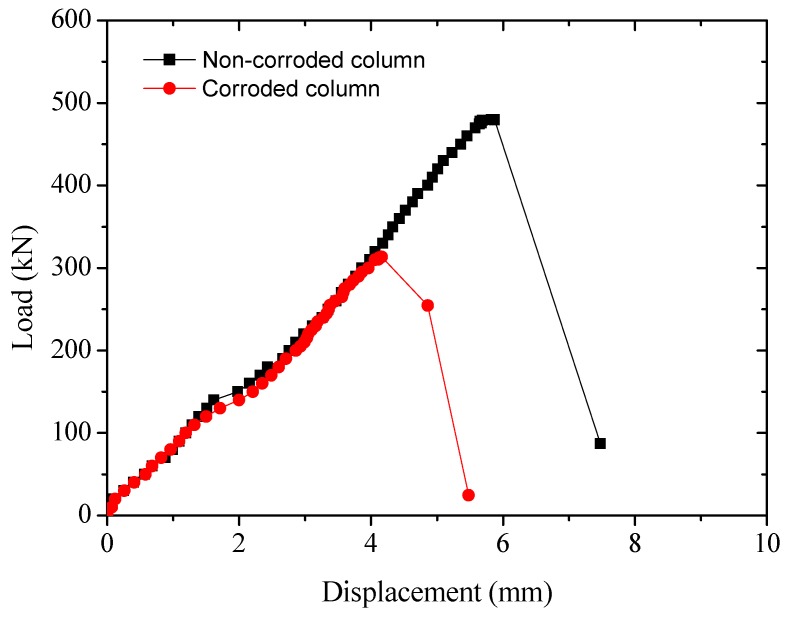
Load-vertical displacement response of RC specimens.

The load-vertical displacement responses of the RC specimens are displayed in Figure 8. It can be seen that the longitudinal displacement of the non-corroded column at failure is greater than that of the corroded column, which is mainly caused by reduction in cross-sectional area of both reinforcements and concrete due to steel corrosion, leading to the reduction of the bearing capacity of the corroded column and accordingly the longitudinal displacement. The failure of both corroded and non-corroded columns shows the typical brittleness modes of a small eccentric column characterized by a sharp drop in load and a further increase in displacement.

#### 3.3.3. Load-Mid-Span Flexural Deflection Response

**Figure 9:**
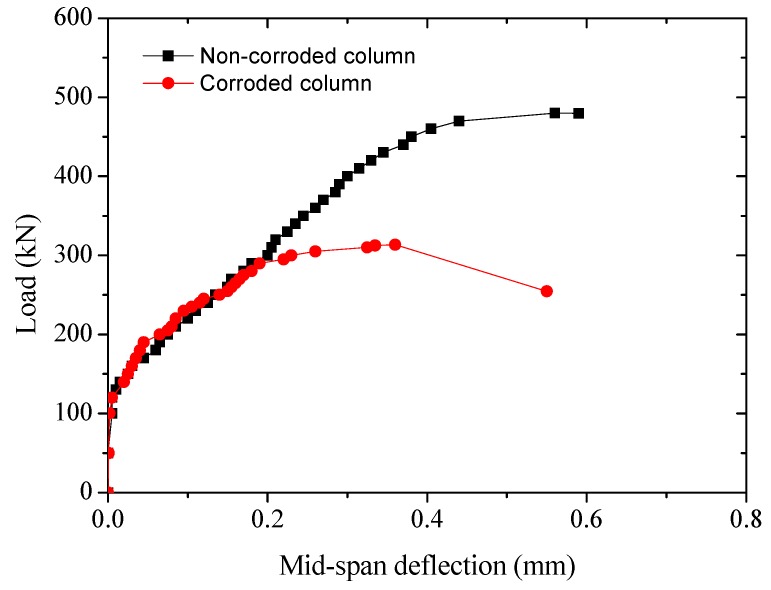
Load-mid-span flexural deflection response of RC specimens.

The load-mid-span flexural deflection responses of the RC specimens are shown in Figure 9. It can also be seen that when the load was small at the initial stage of loading, there was almost no deflection in the mid-span, the phenomenon of which is similar to the axial compression member. When the load was approaching 150 kN, the mid-span lateral deflection began to increase with the increase of the load. Generally, the slope of load-deflection curve of the corroded RC column is smaller than that of the non-corroded RC column.

#### 3.3.4. Load-Strain Response

**Figure 10:**
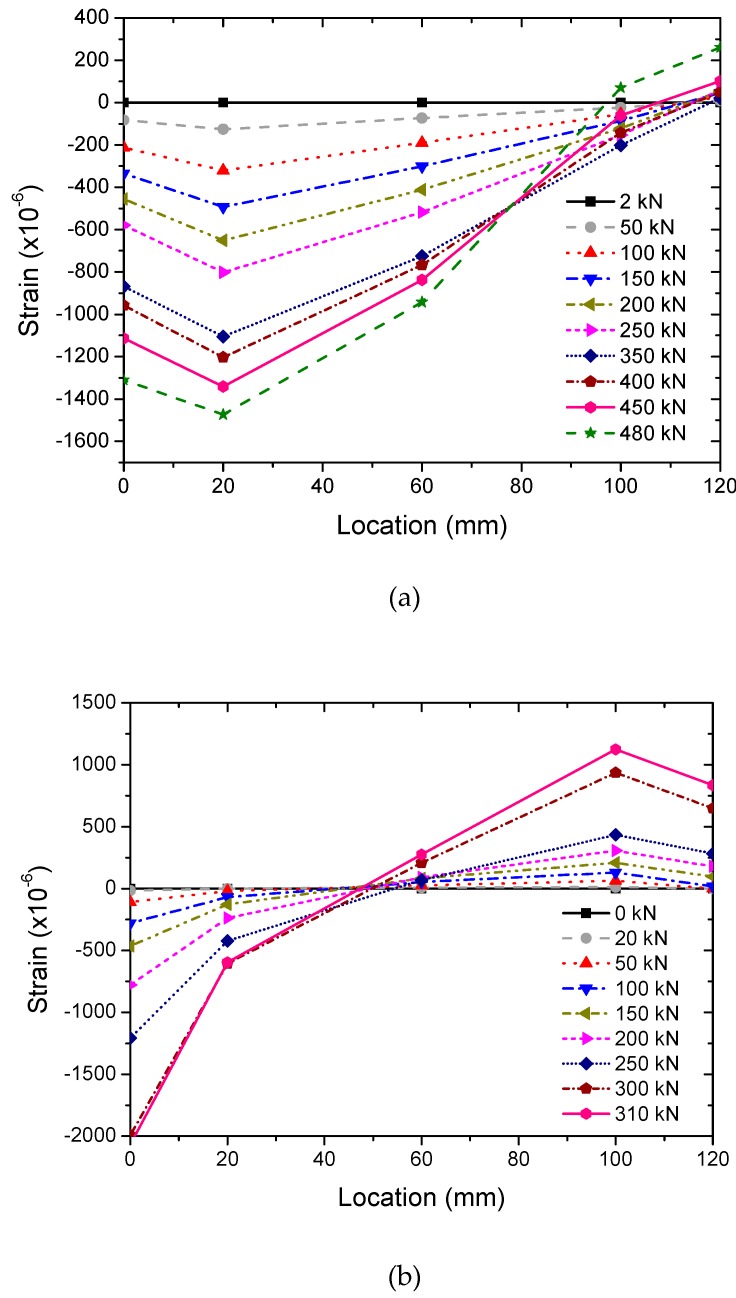
Load-strain response: (**a**) non-corroded column, and (**b**) corroded column.

The load–strain responses of RC specimens are shown in Figure 10. It can be seen from Figure 10 that regardless whether it is corroded or non-corroded, the cross-section compression zone is decreased with the increase of load, and the strain of the concrete in the core zone conforms to the plane section assumption, increased with the increase of load. However, the difference is that under the same eccentricity, most sections of the non-corroded column were under compressive stress, while for the corroded column the compression zone and the tensile zone were almost the same at the initial stage of loading though they changed with the increase of load. This phenomenon indicates that corrosion has a great impact on the mechanical properties of the column. Due to the corrosion of steel bars, the concrete cover was damaged by rust expansion resulting in a reduction of the effective section, which may lead to the possibility of the failure mode shifting from small eccentricity to large eccentricity.

### 3.4. Time-Dependent Development of AE Signal Energy

**Figure 11:**
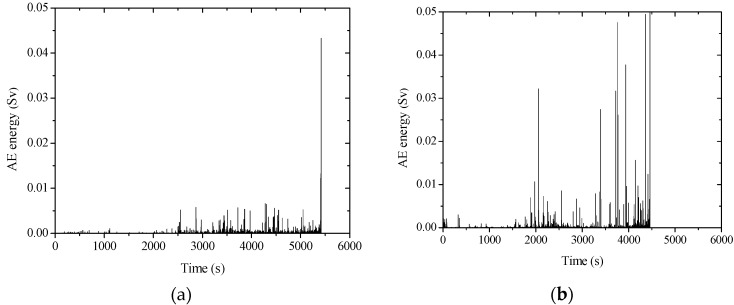
AE energy graph: (**a**) non-corroded column, and (**b**) corroded column.

**Figure 12:**
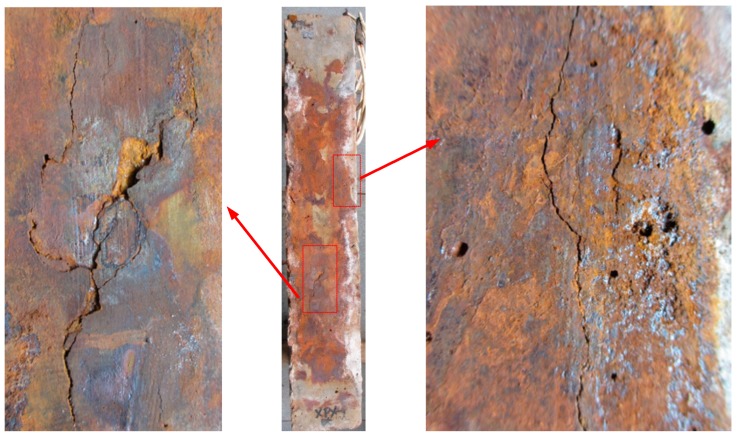
Cracks due to volumetric increase by rust formation on the surfaces of the RC column specimens.

Figure 11 shows the time-dependent development of AE signal energy for non-corroded and corroded columns. The corroded column fails earlier than the non-corroded one at the same loading speed. It can be seen that the non-corroded column has little energy release before 2500 s, and the energy begins to release from 2500 s at a relatively low level until the sudden release happened at around 5400 s. However, the corroded column released some energy before 2000 s, followed by a gradual release, and a final sudden energy release at around 4500 s. This is probably because of the pre-existing internal expansive pressure of corroded reinforcement due to a volumetric increase by forming rusts as shown in Figure 12. It indicates that some amount of energy has already been triggered in concrete internally before external loading is commenced. Therefore, the internally stored energy can be easily released in a manner of crack propagation, even when the external loading level is low. 

**Figure 13:**
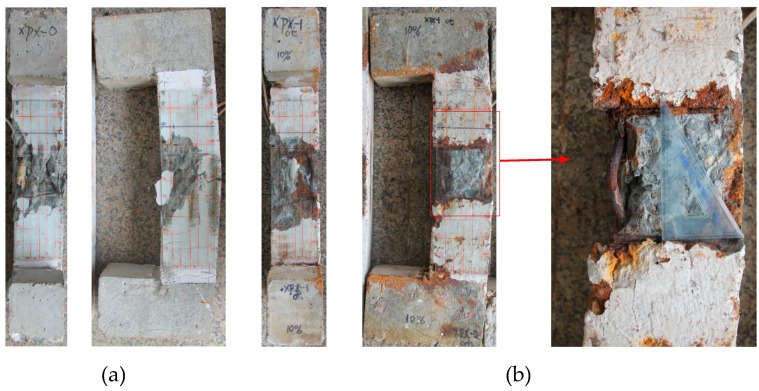
Visual observation of failure characteristics at the surface of RC columns: (**a**) non-corroded column, and (**b**) corroded column.

It can be seen that both corroded and non-corroded RC columns experienced a long time of energy release before failure. However, the corroded column shows more energy peaks, as well as higher intensity of peak energy at failure. It is likely due to different failure modes due to reinforcement corrosion. It was observed that for corroded concrete, not only the concrete in the compression zone was severely crushed, the concrete cover was also peeled off in a large area. By contrast, the non-corroded column did not experience any considerable peeling off of its concrete cover, as can be seen from Figure 13. Therefore, it is reasonable that more energy of the AE signal was released for the corroded column, although the internally stored energy may be larger for the non-corroded column. 

### 3.5. Time-Dependent Development of AE Hits

**Figure 14:**
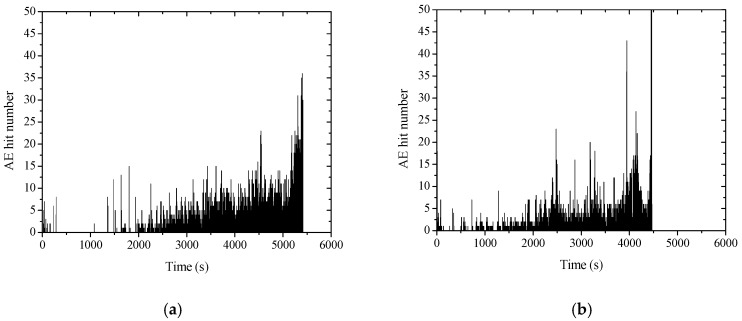
AE ring-down count graph: (**a**) non-corroded column, and (**b**) corroded column.

**Figure 15:**
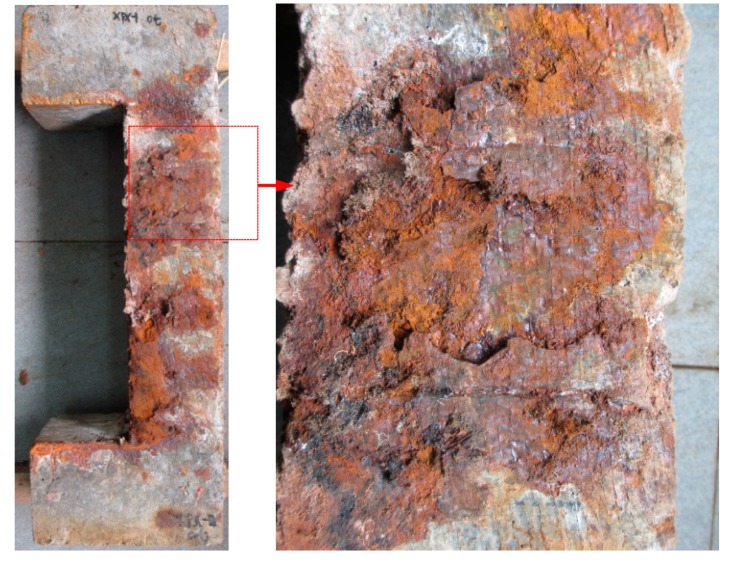
The damage in the RC column specimen caused by corrosion of the reinforcement.

Figure 14 shows the time-dependent development of AE hits for non-corroded and corroded columns. It can be seen that continuous AE hits occurred in the entire loading process of the corroded column. However, in the case of the non-corroded RC column, only a small number of AE hits are observed at the initial stage of the loading. Within the period that AE hits were absent, it may indicate that the non-corroded column specimen experienced elastic deformation and no damage was accumulated at that stage. For the corroded column specimens, the steel corrosion has already caused damage and cracking in concrete, which tend to propagate further due to external loading, as can be seen from Figure 15. 

### 3.6. AE Cumulative Total Hit Number and Cumulative Total Energy

Table 4 summarizes the cumulative AE energy for corroded and non-corroded columns. It can be seen that although with a reduced load-bearing capacity, the total cumulative energy value of the corroded column is significantly larger than that of the non-corroded column. 

**Figure 16:**
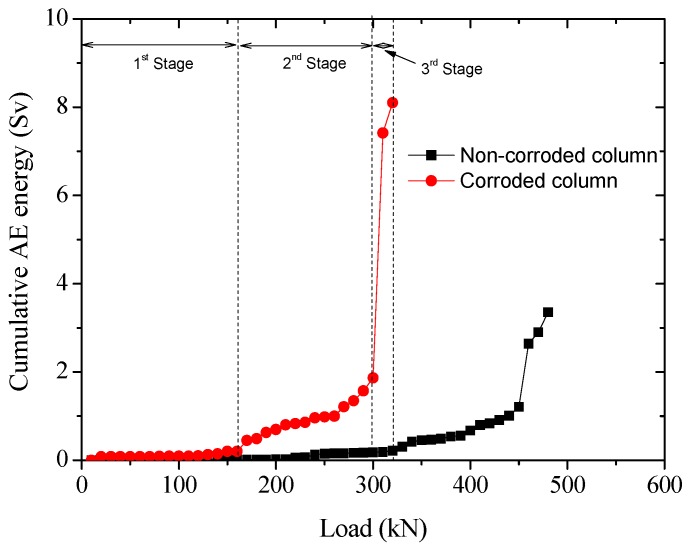
Correlation between AE cumulative energy and loads.

Figure 16 plots the relationship between AE cumulative energy and loads, which can be roughly divided into three stages. The first stage occurs at the early loading, in which the cumulative energy remains at a low level, with a gradual increase of the magnitude of the load. In the second stage, the slope of the curve (i.e., the cumulative AE energy per load applied) is increased. The third stage corresponds to a considerably increased curve slope in which the cumulative energy increased rapidly at almost constant load magnitude. The first stage indicates that the internal micro-cracks propagation is relatively slow with deformation energy stored in the materials; the second stage indicates a progressive accumulation of internal damage; while the third stage indicates the coherence of micro-cracks to macro-cracks and final failure. 

In comparison to the non-corroded column, the slope of the curve in the second stage of the corroded column is relatively high. This is probably due to the additional energy release upon loading due to the pre-existing stress and damage from reinforcement corrosion. In the third stage, the magnitude of suddenly released AE energy of the corroded column is considerably higher than that of the non-corroded counterpart. For the corroded column specimen, as considerable spalling and loss of surface materials occurred in the second stage, the load was mainly carried by the core concrete. As such, the highly intensive AE that signals release in the third stage of the corroded column is related to the crushing of core concrete. 

### 3.7. AE Events Location

**Figure 17:**
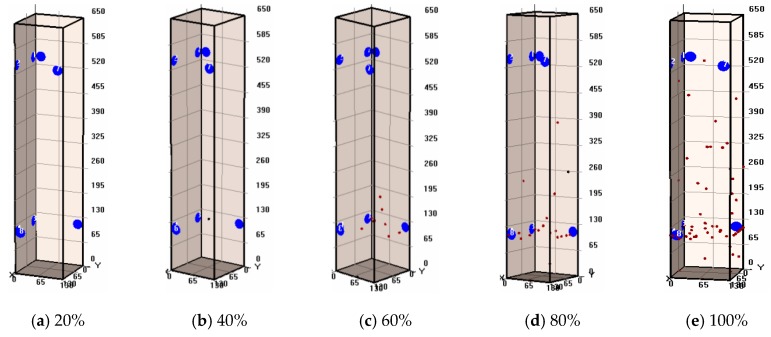
Testing results of AE events location at stages for non-corroded specimens with different load levels (**a**) 20%; (**b**) 40%; (**c**) 60%; (**d**) 80%; (**e**) 100% load levels.

**Figure 18:**
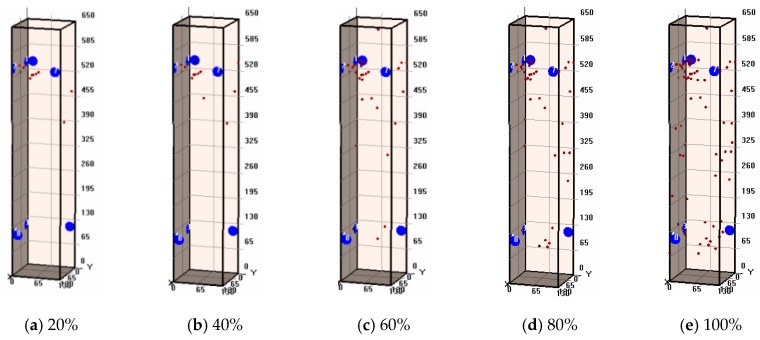
Testing results of AE events location at stages for corroded specimens with different load levels (**a**) 20%; (**b**) 40%; (**c**) 60%; (**d**) 80%; (**e**) 100% load levels.

Figure 17 and Figure 18 show the spatial distribution of AE events of non-corroded and corroded columns under different load levels, respectively. It can be seen that the spatial distribution and evolution of AE events between corroded and non-corroded columns are different. There were more AE events of the corroded column than AE events of the non-corroded column during the whole loading process. Details were illustrated in our previous study [17]. The distinct performance between corroded and non-corroded column is due to the effects of steel corrosion. At the initial loading stage, the pre-existing corrosion-induced micro-cracks can propagate, which is magnified as an AE event. The AE results were in good agreement with the brittle failure mode of RC columns. 

## 4. Damage Evolution Model

AE energy is a crucial index of the instantaneous elastic wave energy of material caused by the rapid release of local strain energy generated by loading. It can be the representative description of AE parameters, as it is closely related to the degree of the damage and the release of energy. Considering that the energy of AE is a macroscopic manifestation of microscopic changes, the damage factor, *D*, can be defined by the relative damage of AE energy, as follows:

(4) $$D=\frac{N\left(V\right)}{N_{P}},$$

in which *N(V)* is the cumulative energy value of AE when the load level is *V* (*V* is defined as the magnitude ratio of the load applied over the ultimate bearing capacity), and *N_P_* is the total AE energy of RC columns at failure.

**Figure 19:**
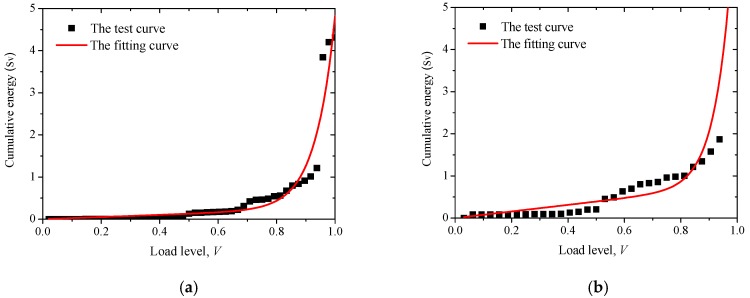
Correlation of load level and accumulated AE energy: (**a**) non-corroded column, and (**b**) corroded column.

Figure 19 shows the correlation between cumulative AE energy and the load level. With an increase of load level, *V*, the cumulative energy of AE increases exponentially. Therefore, the exponential function is proposed to establish the mathematical model of load level and AE cumulative energy value,

(5) N(V)=aV+bexp(cV).

Table 5 gives model parameters of accumulated energy of AE associated with the load level. It can be seen that the accumulated energy fits well with the load level both for corroded and non-corroded RC columns. The correlation coefficient is greater than 0.9, indicating that the model used to characterize the relationship between cumulative energy and load level is reasonable. 

The damage evolution model of RC column can be rewritten as:

(6) $$D=\frac{N\left(V\right)}{N_{P}}=\frac{aV+b\exp\left(cV\right)}{N_{P}}.$$

**Figure 20:**
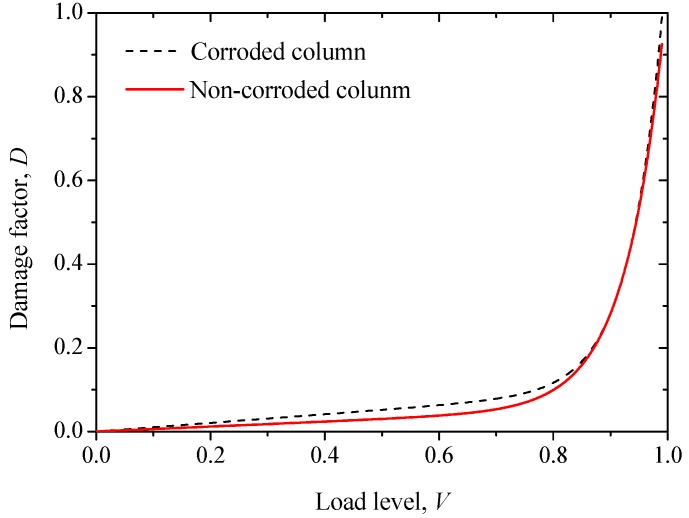
The modeled relationships between the load level and the damage factor for corroded and non-corroded RC columns.

Figure 20 shows the modeled correlation between the load level and the damage factor for non-corroded and corroded RC columns. It can be seen that before the 85% load level, the damage degree is higher in the corroded column, which is in agreement with the test results. Therefore, the damage evolution model of RC expressed by AE energy can be used to describe the corrosion damage of RC columns.

## 5. Conclusions

This paper studies the acoustic emission (AE) characteristics of corroded reinforced concrete columns under eccentric compressive loading. The following conclusions can be drawn:

(1) The AE characteristics of RC columns are in strong agreement with the macroscopic mechanical behaviors observed during the loading and failure process. The reinforcement corrosion considerably affects the mechanical performance of columns, causing the concrete cover to spall during compressive loading.
(2) The presence of corroded rebar makes the AE signals highly active at the initial stage of loading, in comparison to the non-corroded counterpart. Also, a continuous AE hit pattern with a higher number of cumulative hits was observed for the corroded RC column, while the hit event is almost absent at the early loading period for the non-corroded case.
(3) The spatial distribution and evolution of AE events indicate that the reinforcement corrosion considerably affects the initiation, propagation, and cracking evolution in RC columns.
(4) The concrete damage evolution equation presented by AE parameters can quantitatively describe the effects of corrosion damage on the mechanical performance of concrete.

## Figures and Tables

**Table 1:** Mix proportion of concrete.

Water-to-Binder Ratio	Water (kg/m^3^)	Cement (kg/m^3^)	GGBS (kg/m^3^)	Sand (kg/m^3^)	Coarse Aggregate (kg/m^3^)
0.53	203.0	191.5	191.5	766	1149

**Table 2:** Targeted corrosion rate, power, and duration.

Targeted Corrosion Rate (%)	*M_loss_* (g)	*i_corr_* (A/cm^2^)	*A_s,0_* (cm^2^)	*I_corr_* (A)	*t* (h)
10	195.2	0.0002	1238.42	0.2477	754.48

**Table 3:** Actual corrosion rate of reinforcements from gravimetric measurements (%).

Targeted Corrosion Rate	Actual Corrosion Rate		
**10**	**Longitudinal Steel Bars**	Stirrups	Average*
	3.90	12.60	8.25

Note: Average* represents the average weight loss of all reinforcements embedded in the specimen.

**Table 4:** Cumulative AE energy and bearing capacity.

Specimen	Non-Corroded Column	Corroded Column
Cumulative AE energy (Sv)	3.35	8.10
Load-bearing capacity (kN)	480	314

**Table 5:** Parameters for the correlation curve of the load level and accumulated AE energy.

Specimen	*a*	*b*	*c*	Correlation Coefficient
non-corroded column	0.25	1.62 × 10^−6^	14.80	0.93
corroded column	0.78	0.27 × 10^−6^	17.12	0.91
